# Practical and Accurate Indoor Localization System Using Deep Learning

**DOI:** 10.3390/s22186764

**Published:** 2022-09-07

**Authors:** Jeonghyeon Yoon, Seungku Kim

**Affiliations:** Department of Electronics Engineering, Chungbuk National University, Cheongju 28644, Korea

**Keywords:** indoor localization, pedestrian dead reckoning, deep learning, GPS

## Abstract

Indoor localization is an important technology for providing various location-based services to smartphones. Among the various indoor localization technologies, pedestrian dead reckoning using inertial measurement units is a simple and highly practical solution for indoor localization. In this study, we propose a smartphone-based indoor localization system using pedestrian dead reckoning. To create a deep learning model for estimating the moving speed, accelerometer data and GPS values were used as input data and data labels, respectively. This is a practical solution compared with conventional indoor localization mechanisms using deep learning. We improved the positioning accuracy via data preprocessing, data augmentation, deep learning modeling, and correction of heading direction. In a horseshoe-shaped indoor building of 240 m in length, the experimental results show a distance error of approximately 3 to 5 m.

## 1. Introduction

Recently, with widespread use of smartphones, location-based services (LBS) have gained increasing public attention [1,2]. Navigation and route guidance applications that use GPS are the most representative LBS. In addition, LBS for pedestrians, such as surrounding information search services and evacuation route guidance in the event of a disaster, are currently being actively provided. An indoor localization system is an important technology for providing LBS. This enables the estimation of the location of people in a space in which GPS signals are not provided.

Typical mechanisms for indoor localization use wireless signals or inertial measurement units (IMU). Trilateration, triangulation, and fingerprinting methods using the received signal strength indication (RSSI) or channel state information (CSI) have been widely studied in wireless signal-based indoor localization systems. These methods mainly use radio technologies, such as Wi-Fi, Bluetooth, ZigBee, and ultra-wideband (UWB). The wireless signal-based system can operate only in a space with installed infrastructure. In addition, it is difficult to obtain consistent positioning accuracy because the radio signal is greatly affected by nearby obstacles (e.g., walls, columns, and objects) or interference signals. Indoor localization techniques through trilateration and triangulation calculate the position of the target by measuring the distance or angle between the three coordinates that serve as reference points and the tag that serves as the target [3]. In the case of using Wi-Fi as a radio signal, to measure the distance between the access point (AP) and the tag for transmitting the wireless Wi-Fi signal, the RSSI values are converted into distance information or the time of arrival (ToA), and the location is calculated using triangulation. Indoor localization technology through triangulation uses the angle at which radio signals arrive, i.e., angle of arrival (AoA). Mohammed et al. [4] addressed this problem by identifying the condition of an undetected direct path (UDP), which is a straight path from the transmitter to the receiver and introducing a deep learning model to mitigate it. Michael et al. [5] implemented a more accurate time difference of arrival (TDoA) based indoor position recognition technology through multiband (UWB-wideband) UWB technology and a single-input multiple-output (SIMO) method to address the problem of multipath attenuation of radio signals. Shuai et al. [6] proposed enhanced trilateration localization, which improves localization accuracy through quality evaluation and adaptive selection (IT-QEAS). First, they assess the quality of the distance measurement results among the anchor nodes and select the anchor nodes that have the highest quality. Then, a localization calculation was performed with the selected anchor node. However, although indoor localization systems using triangulation and trilateration have improved through various studies, the use of RSSI or CSI is affected by the irregular signal attenuation characteristics of the radio signal depending on the surrounding environment. In addition, because wireless signals are used, APs for transmitting wireless signals must be preinstalled, and there is a possibility of blind spots that radio signals cannot reach, or that radio signals are blocked by obstacles or walls. In particular, localization solutions that need pre-installation in the infrastructure have reduced practicality.

The fingerprinting method divides the service area into several cells, selects a reference location, and receives a wireless signal sent by the Wi-Fi AP at each cell through a smartphone to measure the RSSI values and create a radio map based on it. Subsequently, the user receives a signal from the AP at a random location, measures the RSSI value, contrasts it with a radio map, and estimates the corresponding cell as the user’s location. Yuanchao et al. [7] proposed GradIentFingerprintTing (GIFT) to reduce instability in RSSI values that change over time in indoor environments. Qianwen et al. [8] implemented fingerprinting technology using CSI rather than RSSI and introduced a K-nearest neighbor (KNN) algorithm to increase the accuracy of the final indoor position calculation. Unlike traditional algorithms, Minh et al. [9] introduced a recurrent neural network (RNN) model instead of finding the position of the user one at a time, reducing the instability of the RSSI values that change over time. However, the precise indoor localization of fingerprinting requires a lot of information about the reference locations, that is, cells, used for positioning and the process of collecting RSSI from cells requires service providers to collect them, which is costly. Furthermore, the RSSI value also varies with changes in the surrounding environment as radio maps are pre-written, making it essential to calibrate radio maps over time, and it is impossible to accurately measure RSSI owing to the attenuation of signals caused by interference from adjacent channels. In a relatively static situation, an indoor localization system using fingerprinting can achieve high performance. However, if there are many people and the surrounding environment is very dynamic, it is difficult to expect accuracy because fingerprinting may be hard to implement.

Pedestrian dead reckoning (PDR) is a representative indoor localization method that uses IMU. This uses collected sensor values, such as accelerometers, magnetometers, barometers, and gyroscopes, to estimate the moving distance and direction of movement of people. The positioning accuracy of the PDR is mainly determined by a model or formula that converts the sensor value into moving distance and direction. Various PDR techniques [10,11,12,13,14] have been studied, and they exhibit a position error of several centimeters. However, the models of formulas derived from conventional studies are difficult to generalize under various conditions (e.g., moving patterns, sensor placements, and the surrounding environment). They operate only under specific conditions; therefore, they are difficult to use in practice. PDR is a technology that calculates the next location of pedestrian using sensor data, such as accelerometers, gyroscopes, and magnetometers, from the previous location. The next position of the pedestrian is estimated by estimating the number of steps and stride of pedestrians through acceleration sensors and estimating the pedestrians’ orientation using a magnetometer and gyroscope. The PDR is largely divided into two areas according to how the sensor data are used to calculate the next location. First, the pattern of the data collected by the accelerometer is analyzed to calculate the number of steps. Second, the stride is estimated using the value of the accelerometer. 

For example, the number of steps can be calculated using a peak detection method based on the accelerometer value of a walking pedestrian. With recent improvements in sensor accuracy and computing power of IMU and smartphones, which are data collection devices, indoor location recognition studies using PDR show an error rate within several centimeters. Godha et al. [15] proposed a PDR system that combines GPS coordinates with sensor values obtained using inertial sensors to achieve reasonable accuracy in indoor and outdoor environments. Ionut et al. [16] introduced a practical system that uses an accelerometer and magnetometer from smartphones, without relying on the Wi-Fi’s AP infrastructure, to record pedestrian walking patterns and match them with possible paths on real maps. Kang et al. [17,18] replaced inertial sensors, which are the measuring equipment used in conventional PDR methods, with smartphones to implement indoor position recognition technologies that do not require expensive additional devices or infrastructure. In addition, the number of steps was determined using the accelerometer of the smartphone, and the accuracy of the magnetometer sensor was correlated with the gyroscope to estimate the heading direction. However, it should be considered that the magnetometer sensor is not used as a major data source for estimating the direction of progress for pedestrians because it is greatly influenced by the surrounding environment.

PDR has the advantage that it does not require additional infrastructure. However, there is a significant problem with indoor localization performance, depending on the model and formula that estimates gait information with sensor data. In addition, the selection of the initial position by the user is crucial. The user’s next position obtains results from the prior position, the initial position fades over time because fine errors accumulate in the collected sensor data. Another problem is that indoor localization performance varies depending on the user of the PDR system. Different users have different heights, step lengths, gait patterns, and smartphone placements, which can lead to differences in the performance of the model in estimating the moving speed and heading direction.

The other way of using sensor data to calculate the next position is by using deep learning. Deep learning is a technique for predicting the correct answer by learning patterns or characteristics from training data using models based on neural networks. Because the accelerometer data obtained from a walking person has a certain pattern, it is very suitable for applying deep learning. In other words, the accelerometer value of the pedestrian is composed of a training dataset for learning the deep learning model, and the moving speed or distance of the pedestrian is estimated using the trained deep learning model.

Gu et al. [19] proposed a deep learning-based stride estimation method that can adapt to the characteristics of various users, considering that the walking speed and arrangement state of smartphones are different for each pedestrian. After removing noise from the accelerometer and gyroscope data collected from smartphones using a low pass filter (LPF), the sensor data is trained in a supervised learning model that estimates the stride by dividing the sensor data into segments, each segment representing one step. Kang et al. [20] implemented a new technology for indoor localization, using a deep learning model that learns pedestrian walking pattern data collected outdoors. GPS coordinates and accelerometer data were collected outdoors to determine the moving speed of pedestrians using divided signal frames with hybrid multiscale convolutional recurrent neural network models. In addition, traditional PDR methods are proposed to calculate the number of steps and strides of the IMU signal in a passive way; however, in [20], the moving distance is estimated by calculating the average travel rate from the signal frame.

Although the related studies above have increased the accuracy of indoor localization by applying deep learning, the problem of whether they are practical for pedestrians remains. For example, in [19], when mapping training datasets and labels for learning deep learning models, applicants who collected training data counted their own steps, took them as the ground truth, and organized them into labels. This approach has a very impractical drawback in that the user must calculate the label (ground truth) himself and configure the training dataset until the system shows sufficient performance. To solve this problem, [20] proposed a deep learning model that learns sensor data using GPS as a label for the first time; however, only the steps walked on a straight path were evaluated without considering the orientation of pedestrians.

Because users spend most of their time with their smartphones, the location of their smartphones is highly correlated with their location. In addition, smartphones include Wi-Fi, Bluetooth, and diverse sensors that are widely used for indoor localization [21,22,23]. In this study, we propose a smartphone-based indoor localization system using deep learning. The proposed system operates based on PDR using an accelerometer to estimate the moving speed and a game rotation vector sensor to estimate the heading direction. The data collected by the accelerometer outdoors were used as training data for the deep learning model. The moving speed of the data was labeled using the GPS value. Although the accuracy of the label information decreases owing to the inherent error of GPS, it is very practical because training data can be collected without user intervention. We propose practical solutions and techniques for increasing the positioning accuracy of unspecified smartphone users. The contributions of this study are as follows.

1. Enhancing practicality: Because the moving speed of GPS is used as a data label, anyone can generate training data for supervised learning. The collected accelerometer and gyroscope data were classified according to smartphone placement through unsupervised learning. We derived the required sensor data size for each smartphone placement via empirical experiments and limited the data collection. Using these methods, collecting proper training data for deep learning is possible without user intervention;
2. Improving moving speed estimation: In the data preprocessing step, we removed the noise from the accelerometer and improved the accuracy of the GPS data. Data augmentation using a time-warping scheme expands the collected data size, thereby increasing the accuracy of the deep learning model. Finally, we implemented and compared seven deep-learning models to derive the most accurate model.

The remainder of this paper is organized as follows. Section 2 presents problems that have not been resolved in conventional studies. Section 3 introduces the proposed indoor localization system, and Section 4 evaluates the performance of the proposed system. Finally, conclusions are presented in Section 5.

## 2. Problem Definition

We studied a PDR-based indoor localization using deep learning. In this section, we describe three major problems of conventional PDR-based indoor localization.

### 2.1. Low GPS Accuracy

GPS can provide geolocation and time information to a GPS receiver if four or more line-of-sight GPS satellites exist. Using GPS values as training data labels for deep learning may improve the practicality of the indoor localization technology. However, GPS has inherent errors owing to satellite signal blockage and reflection caused by buildings, trees, etc. If the GPS receiver is located in a city center or forest, the quality of the GPS data may be poor. In this case, using GPS data as training data labels is not appropriate because unreliable labels degrade the accuracy of the deep learning models. This affects the performance of indoor localization systems.

**Figure 1:**
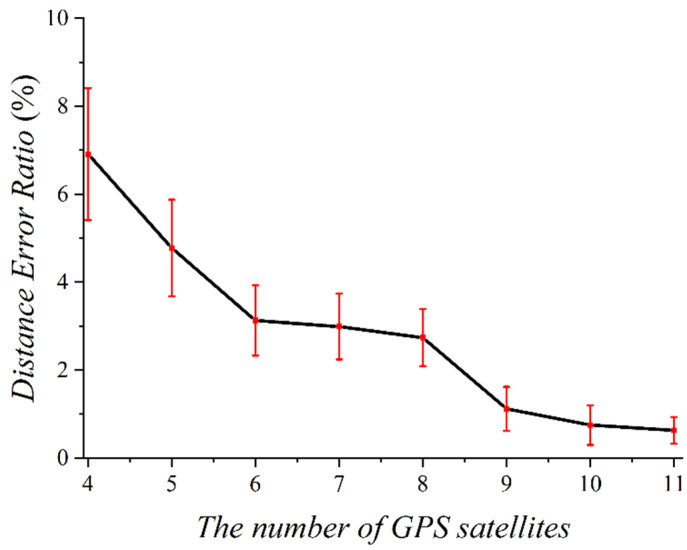
Distance error ratio according to the number of receivable GPS satellites.

We performed an outdoor experiment to analyze the accuracy of the GPS data using a smartphone. The subjects repeatedly moved 75 m in various environments. Figure 1 shows the distance error ratio according to the number of receivable GPS satellites used in the experiment. When the subject moves near high-rise buildings or under a bridge, the smartphone can receive between four and six GPS satellite signals. However, a high distance error ratio of 5.34% to 8.47% was seen in this signal. When the subject moved in the campus playground or field, nine or more GPS satellites could be received. In this case, the distance error ratio was very low (i.e., 0.67–1.72%) compared to the case where the number of receivable GPS satellites is small. In the experiment, we verified that unfiltered GPS data are unsuitable as training data labels for deep learning.

### 2.2. Bias of Sensor Data

An artificial neural network [24] is a statistical learning algorithm constructed by imitating the manner in which nerve cells or neurons are inter-connected in the human brain. Just as human neurons receive and store input values through synapses from several other neurons and then export output values to the next neuron, the artificial neural network model neuron receives multiple inputs and transmits output values to the next neuron in the model. Artificial neural networks are constructed by stacking several neuron models. Deep learning is a type of machine-learning algorithm based on artificial neural networks. Deep learning has been widely used in various applications.

**Figure 2:**
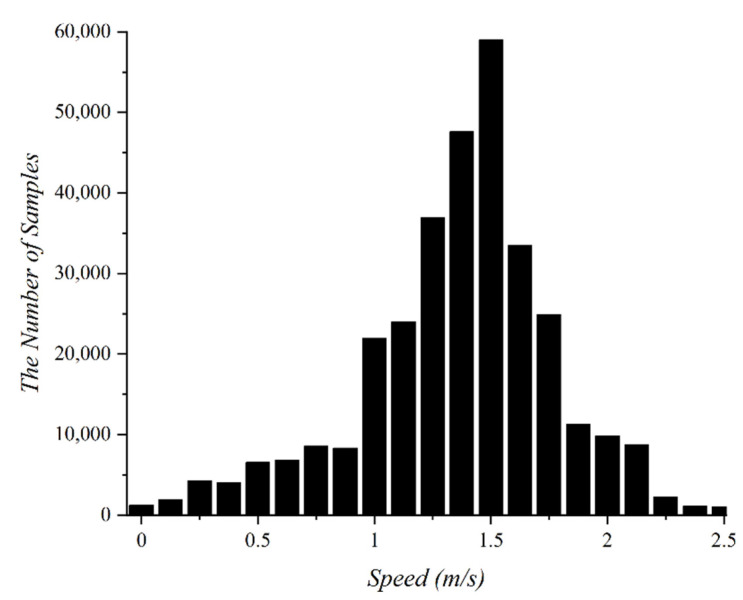
Distribution of the collected data according to moving speed.

Deep learning refers to a learning model that predicts outcomes by extracting features or patterns from training data. The size and quality of the training data significantly influence the performance of the deep learning model. Thus, it is very important to secure a sufficient amount of high-quality data from which a deep learning model can extract features. A PDR-based indoor localization system mainly uses sensor values, such as data from accelerometers, magnetometers, barometers, and gyroscopes for training. To determine the patterns of movements in daily life, we conducted an outdoor experiment to collect data. In the experiment, a subject collected sensor values every second using a smartphone, while walking freely for 3 h. The moving speed of the subjects was obtained using GPS. Figure 2 shows the distribution of the collected data with respect to the moving speed. As a result, 66.3% of the collected data were distributed between the moving speeds 1.3 m/s and 1.7 m/s. Most of the collected data were within the average human walking speed. Since the number of samples for the speed range 1.3–1.7 m/s is much larger than that for other speeds, the data is biased. If these biased data are used for training deep earning models, it is difficult to expect high accuracy in the case the subject is not walking at an average speed. We do not need to consider data bias, if we can collect huge amounts of data. However, because collecting data is expensive, we assume that data collection is minimized.

### 2.3. Effect of Magentometer

The heading direction estimation of pedestrians for the indoor localization system is as important as the estimation of the moving distance. Many conventional studies on heading direction estimation use a magnetometer to measure the magnetic north. However, the magnetometer reacts sensitively to the surrounding environment, such as walls, obstacles, and steel, resulting in errors that cannot correctly measure the magnetic north. Android smartphones provide the absolute heading direction through the rotation vector sensor by combining the data obtained from the accelerometer, magnetometer, and gyroscope. Further research is required to estimate a stable heading direction without using a magnetometer that is affected by the surrounding environment.

## 3. Indoor Localization System

**Figure 3:**
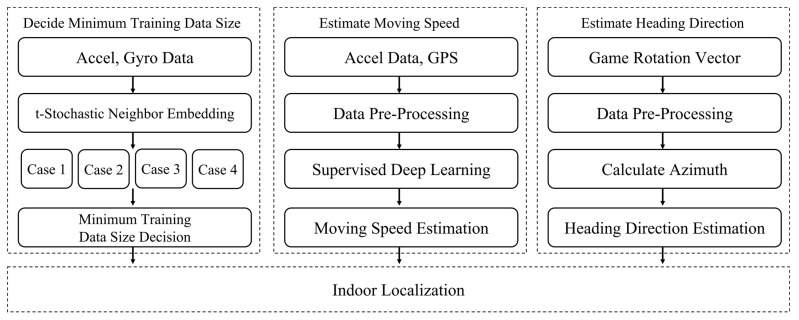
Indoor localization system overview.

In this section, we introduce a new indoor localization system for pedestrians that uses smartphones. Figure 3 illustrates the structure of the smartphone-based indoor position recognition system using the deep learning method proposed in this study. The proposed system consists of three steps: (1) classifying smartphone placements; (2) estimating the moving speed using deep learning, and (3) estimating the heading direction using game rotation vector sensors. The detailed operation of each step is described in the following subsection.

### 3.1. System Overview

The first step was to classify the accelerometer and gyroscope data of pedestrians collected through smartphones as smartphone placements. There are many cases of smartphone placement; however, we assume only the four most representative cases. If it is possible to classify the collected data as smartphone placements, pedestrians can check the amount of data they collect in real-time. In addition, when sufficient pedestrian speed data, enough to achieve good performance, has been collected, the system stops collecting data to minimize battery consumption in smartphones.

The second step involved moving speed estimation using supervised learning. The 3-axis accelerometer data collected from the pedestrians’ smartphones and the moving speed labels recorded every 1 s through GPS comprise the training datasets. Before constructing the training dataset, the accelerometer and GPS data were pre-processed. Since the 3-axis accelerometer data change as the smartphone’s placement and tilt, regardless of these changes, acceleration data are collected with 3-axes fixed. In addition, accelerometer data are filtered through Kalman and low-pass filters. These filters minimize the impact of the drift of the accelerometer and sudden environmental changes. Because the labels of the deep learning model require high accuracy and reliability, only those GPS coordinates are used as labels and used to calculate the moving speed for which there are more than nine satellite signals that smartphones can receive. As explained in Section 2.2, most of the data were within the average walking speed. To eliminate this bias, additional data on various walking speeds were obtained using time-warping data augmentation technology. The training dataset obtained after these processes is used as an input to a deep learning model that estimates the moving speeds of pedestrians. To find the optimal deep learning model for estimating moving speed, we evaluated the accuracy of moving speed estimation by implementing seven supervised learning models.

In the third step, the heading direction of pedestrians is estimated. Magnetometers are extremely sensitive to the surrounding environment and may have errors, which will affect the estimation of the heading direction. Therefore, we estimated the heading direction using game rotation vector sensors that do not include magnetometers.

### 3.2. Smartphone Placement Classification

This section describes the technology used for classifying the collected data according to the placements in which pedestrians hold their smartphones. To classify the collected data according to each case, t-stochastic neighbor embedding (t-SNE) [25] unsupervised learning was used. t-SNE is a technique that classifies and visualizes high-dimensional vectors into 2- or 3-dimensional maps humans can understand, while preserving the distance between the data. The t-SNE unsupervised learning model uses accelerometer and gyroscope data as training datasets, and smartphone placements as labels (i.e., cases). Additional gyroscope values were used to increase the accuracy of the classification. Gyroscopes are suitable for data classification because the change in the value, which depends on the placement of the smartphone, is greater than that of the accelerometer. Although the accuracy should be evaluated according to all possible smartphone placements, this study assumes only four of the most commonly used. The four cases are as follows: (1) hand-held, (2) hand swing, (3) in pocket, (4) in a backpack. In this step, pedestrians can check the size of the collected data for each case in real time. In our empirical experiments, we found the proper data size to be 3 h per case (i.e., a total of 12 h) to ensure sufficient accuracy to estimate the moving speed. Thus, the data collection process stopped when the data size was at least 3 h and a total of 12 h to prevent excessive smartphone battery usage. This method allows pedestrians to know the minimum size of the training dataset for learning the deep learning model, thus gaining the practicality that battery consumption can be reduced in the data collection process.

### 3.3. Moving Speed Estimation

The moving speed estimation step consists of data pre-processing and a supervised learning-based deep learning model. The data needed to estimate the moving speed were the GPS and accelerometer values collected outdoors. Accelerometer data were used to extract the features of pedestrian gait patterns and were used as training datasets for the deep learning models. The moving speed calculated via GPS coordinates was used as a label for the training datasets as in [20].

**Figure 4:**
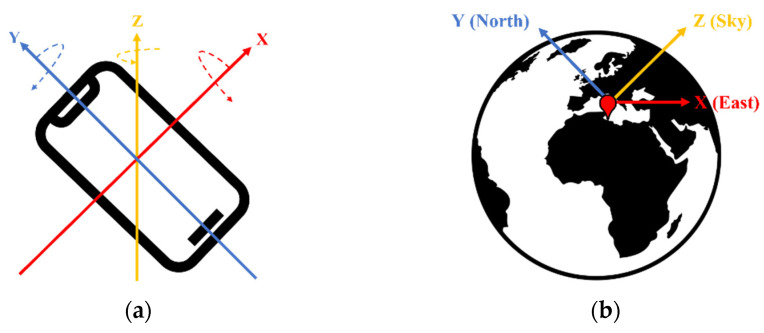
Coordinate system (**a**) Device oriented coordinate (**b**) Earth oriented coordinate.

The accelerometer data collected from the smartphones were pre-processed for use as input to the supervised learning model. The accelerometer data provided by the smartphone were device oriented. As can be seen in Figure 4, when the data are collected by device orientation, there is a problem in that the data show different characteristics depending on the placement and orientation of the smartphone from which they were collected. This means that even when moving in the same direction, the data values of the collected accelerometer vary depending on the tilt and placement of the smartphone. Because pedestrians can place the smartphone anywhere near the body, data should be collected with respect to three axes that are fixed regardless of the tilt and placement of the smartphone. To solve this problem, data pre-processing is used to convert the raw coordinates of the accelerometer (i.e., device-oriented coordinates) into earth-oriented coordinates. Alwin et al. [26] presented a method for converting device-oriented 3-axis accelerometer values into earth-oriented coordinates by multiplying them with rotation vector sensor values. The following Equation (1) represents a formula for transform the device-oriented coordinate to the earth-oriented coordinate.

(1) $$\left[\begin{array}{c} A_{x}\\ A_{y}\\ A_{z} \end{array}\right]=R{\left[\begin{array}{ccc} a_{x} & a_{y} & a_{z} \end{array}\right]}^{T}$$

The R in the right term from Equation (1) represents the transform of the rotation vector value obtained through the rotation vector sensor into a rotation matrix, and $a_{x},$ $a_{y}$ and $a_{z}$ represents the raw value of accelerometer. Both values are obtained through smartphones. Each of $A_{x}$, $A_{y}$ and $A_{z}$ in the left column represent the value of the three axes of acceleration converted to the earth reference coordinate system. Using this method, accelerometer values can be converted into earth-oriented coordinates to collect data, regardless of smartphone placement. The authors used Kalman filters and low-pass filters for the accelerometer data. Filtering accelerometer data eliminates drift and noise from smartphones.

In this paper, we use the pedestrian moving speed obtained through the GPS as a label for a deep learning model. Labels are correct answers to training data that are input to deep learning models, which affect the performance of deep learning models depending on the accuracy of the labels. In [20], the accuracy of the label is relatively low, because the moving speed was calculated and used as a label regardless of the reliability of GPS. Therefore, in this paper, to increase the reliability of labels on training datasets, we compose the training datasets with only the data collected when the receivable number of satellite signals on smartphones is more than nine. This is a new and novelty method that has not been considered so far. The GPS error rate is inversely proportional to the number of satellite signals that a smartphone can receive, as shown in Figure 1. If the number of satellite signals that can be received is more than nine, high reliability of the label can be expected because the GPS measurement accuracy is approximately 98%. We calculated the moving speed for one second using the GPS coordinates when more than nine satellite signals were received, while collecting the sensor data. Equation (2) represents a formula for obtaining a moving speed of a pedestrian through a distance obtained by collecting GPS coordinates every second.

(2) Pedestrian Moving speed=distance÷1

The calculated moving speed and accelerometer values were mapped for a period of one second and used as training data for the supervised learning model. This process can solve the reliability problem for labels collected in the GPS coordinates presented in Section 2.1.

**Figure 5:**
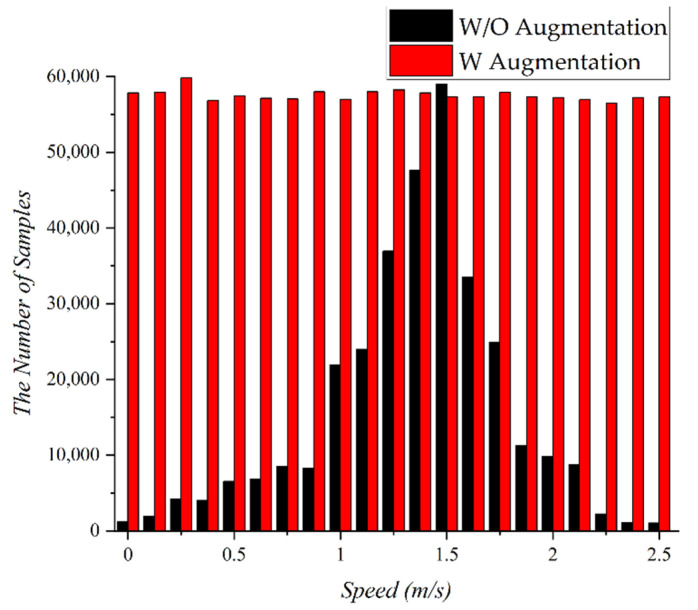
Original (black) and time-warped (red) moving speed data.

As shown in Figure 2, most of the data collected at the average moving speed are concentrated at a particular moving speed. If the training data do not have sufficient data for all moving speed labels, the deep learning model will fail to estimate the exact moving speed if the pedestrian walks slower or faster than the average moving speed. Thus, to eliminate bias in the collected data, we introduced time-warping to augment the amount of data for various moving speeds. Time warping is a transformation that allows each element value in a sequence to be repeated by an arbitrary number and can compress or expands fixed-length time series data to a particular length. For example, two sequences $S=\left\{20,20,21,20,23\right\}$ and $Q=\left\{20,21,21,20,20,23,23,23\right\}$ may be converted into the same sequence $A=\left\{20,20,21,21,20,20,23,23,23\right\}$ by time warping. The distance between the two sequences after time warping is defined as a time warping distance. Figure 5 shows the results augmented to data of 24 h by time-warping the original data of 3 h. The black bar graph represents the original data, and the red bar graph represents the data enhanced through time-warping. By augmenting the data through time warping, the data can be constructed to have an even distribution at various moving speeds rather than being concentrated at a specific moving speed. This process solves the data-bias problem presented in Section 2.1.

**Figure 6:**
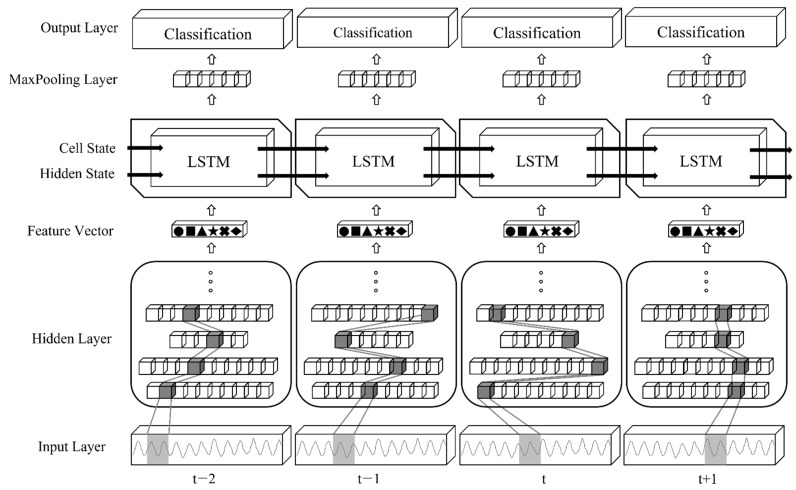
LSTM-C Model.

The data of 12 h are augmented to a size of 96 h through time warping and are used as a training dataset for supervised learning-based deep learning models. The deep learning model derives a result by extracting patterns or features from the accelerometer data of the training dataset and estimating the moving speed for a one-second data segment when learning is completed. Estimation accuracy is the most important indicator of the performance of the deep learning model. One representative way to improve the accuracy of the model is to use an appropriate deep learning model. In this study, performance was evaluated by implementing seven deep learning models: CNN [27], GRU [28], LSTM [29], C-GRU, C-LSTM, GRU-C, and LSTM-C, to find the optimal deep learning model with the highest moving speed estimation accuracy. These models are explained below. A convolutional neural network (CNN) is a useful supervised learning model for identifying patterns to recognize images. CNNs are mainly used to find and learn patterns from training data and then classify new images using learned patterns. It is widely used in object recognition fields, such as self-driving cars and facial recognition. In contrast, the recurrent neural network (RNN), represented by long short-term memory (LSTM) and gated recurrent units (GRU), is a supervised learning model that has strengths in processing sequence data, such as time series and natural language. Because sequence data cannot understand the entire context simply by knowing and understanding only one data segment, LSTM and GRU are models that have improved existing RNNs to identify and learn the context of these sequence data. LSTM and GRU are used in various fields, such as voice recognition and sentence arrangement from listed words. Convolutional GRU (C-GRU) and convolutional LSTM (C-LSTM) refer to deep learning models in which CNN and RNN are combined and are called convolutional recurrent neural networks (CRNNs). Given location and shape data, such as images, the correct answer is estimated by first extracting features from the CNN layer and inputting these features in chronological order into the RNN layer. That is, it is a model that extracts and utilizes time series data from image data. GRU convolutional (GRU-C) and LSTM convolutional (LSTM-C) extract features or patterns from data through convolutional layers and input them into the RNN layer in the same manner as CRNN, but the final output process is different. In the case of the CRNN, the correct answer is estimated by entering the output value of the RNN into several dense layers. However, in the case of LSTM-C and GRU-C, the output value of the RNN is input to one max pooling layer so that the output value of the layer appears as the final estimate. Figure 6 shows the model configuration diagram of the LSTM-C.

### 3.4. Heading Direction Estimation

In the heading direction estimation step, the exact heading direction of the pedestrians was measured. Android smartphones use a rotation vector sensor, including an accelerometer, gyroscope, and magnetometer, to measure the orientation of smartphones [30]. However, obstacles, walls, and steel can affect the magnetometer of the rotation vector sensor, so it can measure the wrong orientation. The other sensor for measuring the orientation is the game rotation vector sensor [31] provided by Android. It is identical to the rotation vector sensor in that it uses an accelerometer and a gyroscope, but it does not use a magnetometer. Hence, the game rotation vector sensor does not provide an absolute heading direction, that is, the magnetic north. Its *y*-axis represents the upward direction of the smartphone, not the north. Therefore, we used game rotation vector sensors instead of rotation vector sensors to measure the orientation. The game rotation vector is a combination of angles and axes in which the smartphone rotates by *ψ*, *θ*, and *ϕ* around the *x*-, *y*-, and *z*-axes, indicating the orientation of the device, as follows:

(3) $$x\left(\psi \right)=x\cdot \sin\left(\frac{\psi }{2}\right)$$

(4) $$y\left(\theta \right)=y\cdot \sin\left(\frac{\theta }{2}\right)$$

(5) $$z\left(\phi \right)=z\cdot \sin\left(\frac{\phi }{2}\right)$$

The three-axis vector value obtained by the game rotation vector sensor is converted to a rotation matrix using quaternion through the getRotationMatricFromVector (getRMFV) function. Subsequently, the getOrientation function allows the rotation matrix acquired from the getRMFV function to be represented by the placement state of the smartphone. Three values are derived. The first value is the azimuth, the angle of rotation on the negative *z*-axis and represents the angle between the *y*-axis of the device and the Earth’s Arctic point. The second value is the pitch, which is the angle of rotation on the *x*-axis, and represents the angle between the plane parallel to the screen of the smartphone and the plane parallel to the ground. The third value is the roll, that is, the angle of rotation on the *y*-axis, which represents the angle between the plane perpendicular to the ground and the plane perpendicular to the smartphone screen.

(6) $$q1=rotationVector\left[0\right]$$

(7) $$q2=rotationVector\left[1\right]$$

(8) $$q3=rotationVector\left[2\right]$$

(9) $$q0=1-\left(q1*q1\right)-\left(q2*q2\right)-\left(q3*q3\right)$$

The Rotation Vector [0], [1], and [2] are rotation vector values indicating the tilt of the smartphone same as Equations (3)–(5), respectively. Equations (6)–(9) represent quaternions used to transform the rotation vector value as a rotation matrix. The heading direction of pedestrians can be obtained from the azimuth, and the formula is as Equation (10). The ψ, θ and ϕ each represent an angle at which the smartphone is rotated with respect to the *x*-, *y*-, and *z*-axes, and the angle can be obtained by the rotation vector sensor.

(10) $$\mathrm{Azimuth}=\tan^{-1}((\cos\phi \sin\psi +\sin\phi \cos\theta \cos\psi )-(-\sin\phi \cos\psi -\cos\phi \cos\theta \sin\psi ))\;$$

**Figure 7:**
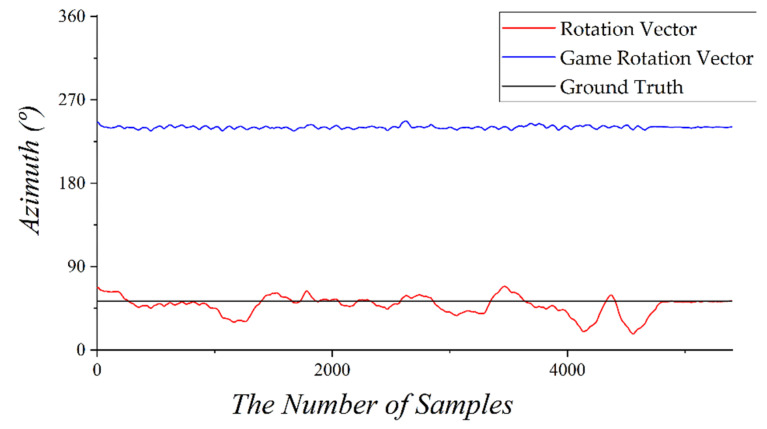
Comparison of each azimuth through rotation vector sensor and game rotation vector sensor.

Figure 7 shows a graph measuring the azimuth through a rotation vector sensor and a game rotation vector sensor by walking through a straight passage in a building. The black line represents the actual azimuth measured from the north using Google Maps. Because the rotation vector sensor includes a magnetometer, it is difficult to measure a stable azimuth owing to a door or an obstacle made of iron and even an empty space. However, the result of measuring the azimuth angle using the game rotation vector sensor was very stable. Because the azimuth is measured using only accelerometers and gyroscopes, excluding the magnetometer, the results were very similar to the ground truth, except for the shaking caused by the walking of pedestrians.

## 4. Performance Evaluation

### 4.1. Evaluation Environments

**Figure 8:**
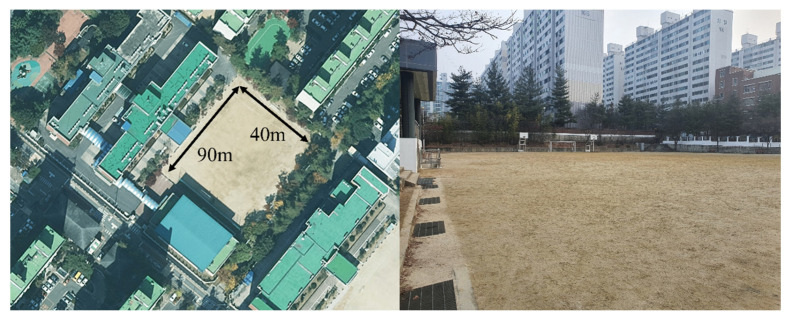
Outdoor data collection environment.

**Figure 9:**
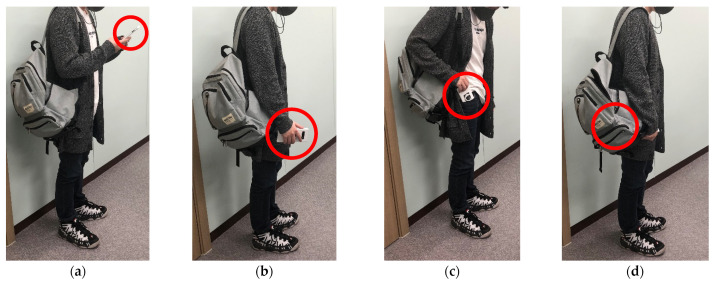
4 data collect situations (**a**) hand held (**b**) hand swing (**c**) in pocket (**d**) back pack.

We evaluated the performance of the proposed system using a computer with the following specifications: Intel i5-6500 CPU, 40 GB RAM and Geforce RTX 2080 GPU. Datasets for supervised and unsupervised learning models were collected through the following processes: As shown in Figure 8, the training data were collected from the playground in Yongam Middle School in Sangdang-ku, Cheongju city, which was approximately 40 m × 90 m area, and had no obstacles or walls. We collected data for four situations. First, hand-held; second, hand-swing; third, in-pocket; and fourth, in a backpack. We collected training data over 10 h for each orientation, for a total of 40 h, as shown in Figure 9. The sampling rate of each sensor is 100 Hz, and the sensors used in this system are an accelerometer, gyroscope, GPS, and a game rotation vector sensor built into the Samsung Galaxy s10. Next, the conditions for collecting data for the validation data set were the same as the training data, except that it was collected inside the E10 building of Chungbuk National University. Table 1 presents the hyperparameters of seven deep learning models implemented to identify which model(s) are optimal for the indoor localization systems proposed in this paper.

### 4.2. Results of Smartphone Placement Classification

**Figure 10:**
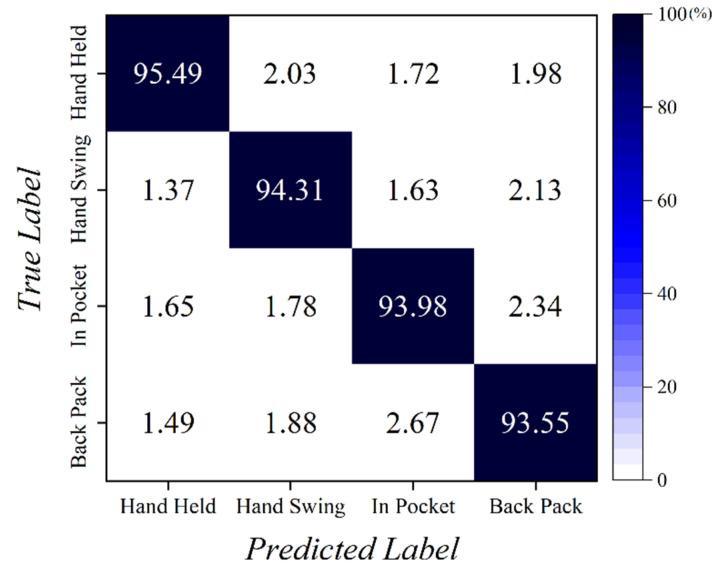
Smartphone placement classification result.

Figure 10 shows the confusion matrix of the results from the t-SNE unsupervised learning model. The model was trained using a training data set of 40 h consisting of accelerometer and gyroscope data. The graph resulted from the validation data set of 2 h. The *x*-axis of the Figure 10 represents the results predicted by the unsupervised learning model, and the *y*-axis represents the case in which the actual data belong, that is, the label. For example, in the hand-held case, out of 7200 (half-hour data) samples, the model predicted 6875 data correctly as be hand-held (95.49%). For the remaining 325 data the model failed to classify them as hand held and classified into one of three other cases. Figure 10 indicates that unsupervised learning using t-SNE can sufficiently classify the data collected in each case.

### 4.3. Results of Moving Speed Estimation

Table 2 shows the results of augmenting data of 12 h to 96 h with time-warping, using them to train seven models, and evaluating the accuracy of each model in estimating the moving speed with a validation data set of 2 h. Of the seven supervised learning models, the best performing model was the LSTM-C model, with approximately 95.6% accurate moving speed estimation. Therefore, the deep learning model to be used in the indoor localization systems proposed in this study was determined to be LSTM-C.

**Figure 11:**
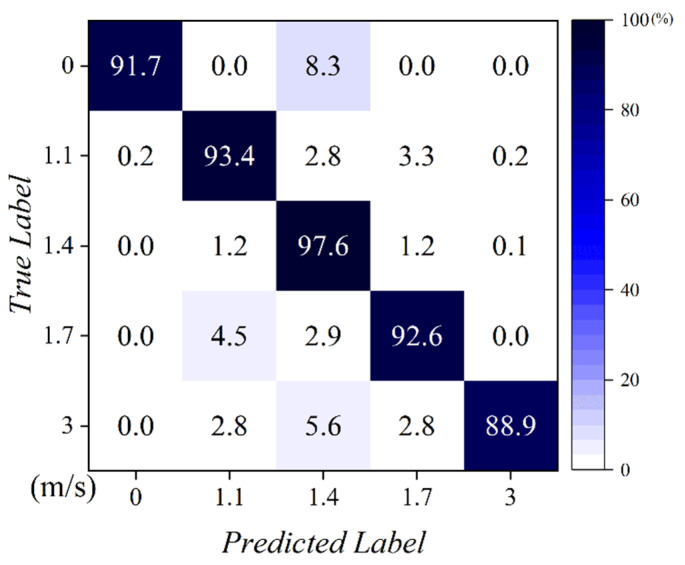
Moving speed estimation using LSTM-C.

Figure 11 shows the confusion matrix graph of the movement speed estimation of the LSTM-C model. The LSTM-C model was trained using a non-augmented training data set of 40 h. As shown in the Figure 11, the LSTM-C model estimated the data for 1.4 m/s with a high accuracy of 97.6%. However, data belonging to labels other than 1.4 m/s showed relatively low accuracy. These results show that most of the training dataset is densely distributed around 1.4 m/s, as shown in Figure 2, and that the model has not learned enough features for other moving speed labels to classify them correctly. Accordingly, in this study, the training dataset was augmented with time warping to obtain an even distribution across various moving speed labels.

Table 3 shows the results of the estimation accuracy of the LSTM-C model, trained by the various training datasets augmented with time warping for moving speed. The TW class represents the number of classes in which the original data is augmented using time-warping. For example, if the TW class is one, the training data are not augmented, if the TW class is three, the training dataset is augmented to three classes, including the original data. With the original data without augmentation, sufficient moving speed estimation accuracy was not achieved. When the TW class was 3, sufficient accuracy in estimating the moving speed for the original data of 16 h and 20 h was observed. However, by checking the loss graph, we found an overfitting problem and did not see high accuracy for various moving-speed labels. Using a confusion matrix, as shown in Figure 11, it was found that the accuracy of the approximately 1.5 m/s label was sufficiently high, but similar accuracy could not be achieved for the other moving speed labels. When the TW class was 5 or 7, sufficient accuracy was achieved, and overfitting problems did not occur. However, the data were not augmented for various moving speed labels; therefore, the model did not show a high accuracy for all labels, as shown in Figure 11.

However, when the TW class was more than nine, including the original data, the model showed high accuracy for all labels. If the original data are augmented to more than nine TW classes, the indoor localization system proposed in this study can obtain a high moving speed estimation accuracy for all moving speed classes. However, the larger the total size of the training data set, created by increasing the number of TW classes, the smaller is the increase in accuracy, and the amount of data increases rapidly. For example, the data augmented with 5 and 13 TW classes from 12 h of original data were 60- and 156 hours, respectively, but achieved 95% and 96% accuracy, respectively. In this case, the amount of data increased significantly; however, the accuracy increased only by less than 1%. Of course, it is better to augment and learn the original data into 13 TW classes from the perspective of performance, but this is not practical from a system user perspective. The indoor localization system proposed in this paper categorizes the collected data using unsupervised learning, augments the training dataset with time warping and then learns through the LSTM-C model. These processes require a great deal of time, especially the process of augmenting with time-warping and performing learning of augmented data with deep learning models; the larger the dataset, the more time it takes. Therefore, in this study, the minimum size of the original data required for learning the deep learning model parameters was limited to 3 h for each case, i.e., a total of 12 h, and the number of TW classes was set to nine, including the original data.

**Figure 12:**
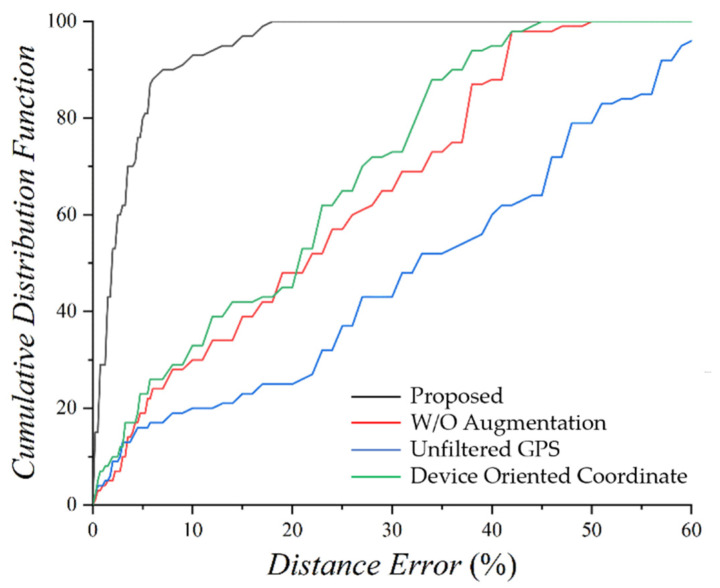
CDF graph of moving speed estimation.

Figure 12 shows a CDF graph of the moving speed estimation error rate of an LSTM-C model that learns the training data set augmented to 96 h and applies the improvements proposed in this paper for three different comparators. The red line is a CDF graph of the error rate for estimating the moving speed of the original data of 12 h without augmentation. A marked improvement in accuracy, compared to the other lines, can be seen for the black line, which is the result of applying all the improvements proposed in this study. Furthermore, as shown in Figure 5, time warping eliminates data bias, which increases the estimation accuracy performance for various moving speeds. The blue line is a CDF graph of the moving speed estimation error rate for the test data of 2 h after learning the LSTM-C model by augmenting the collected data of 12 h into nine TW classes, regardless of the number of satellites, to evaluate the reliability of the ground truth information. If data are collected regardless of the number of satellites, the blue line in Figure 12 shows that the exact moving speed cannot be calculated accurately because of the lack of reliability in the moving speed labels, that is, the ground-truth information required for the supervised learning model is not reliable. However, the data collected when the number of satellites is greater than nine are relatively high in accuracy for the moving speed label and show a higher performance. This means that the higher the reliability of the previously mentioned ground-truth information, the better are the deep learning model results. The green line is a CDF graph of the performance of the training dataset collected using device-oriented coordinates. If the data are collected by the device-oriented coordinate system, the sensor value depends on the *y*-axis of the smartphone. However, when data are collected through an earth-oriented coordinate system, the sensor value does not change depending on smartphone placement. For example, when a smartphone is freely placed at a moving speed of 1.5 m/s for 10 s, the data collected into a device-oriented coordinate system shows significant variations depending on the placement of the smartphone. That is, the 3-axis accelerometer sensor varies with each change in the smartphone placement, but for the earth-oriented coordinate, the values of the 3-axis of the collected accelerometer values do not change even if the smartphone placement changes. It can be seen from the green line in Figure 12 that the device-oriented coordinate data show a large error compared to the data collected through the earth-oriented coordinate. This means that the process of generalizing training data has a significant impact the deep learning model outcome and that the indoor localization system proposed in this paper, which transforms the sensor data collected into earth-oriented coordinates and performs the generalization process and produces improved results.

### 4.4. Results of Indoor Localization System

This section evaluates the performance of path estimation of indoor localization systems by collecting test datasets for routes, including straight paths and rotations in indoor environments. Table 4 lists different versions of the training datasets for evaluating the performance of indoor localization systems. For example, the ‘Without augmentation’ training dataset refers to a dataset that is composed of data transformed the device-oriented coordinate to the earth-oriented coordinate when the receivable number of satellite signals is more than nine but is not augmented by time-warping. Another example is the ‘Unfiltered GPS’ training dataset, which is composed of data that transformed the device-oriented coordinate to the earth-oriented coordinate and is augmented by time-warping but is composed in all situations, regardless of the receivable number of satellite signals. Finally, the ‘Device oriented coordinate’ training dataset that is composed of data collected when the receivable number of satellite signals is more than nine and augmented by time-warping but does not transform the device-oriented coordinate to the earth-oriented coordinate. On the other hand, the ‘Proposed’ training dataset refers to a dataset with all the techniques proposed in this paper.

**Figure 13:**
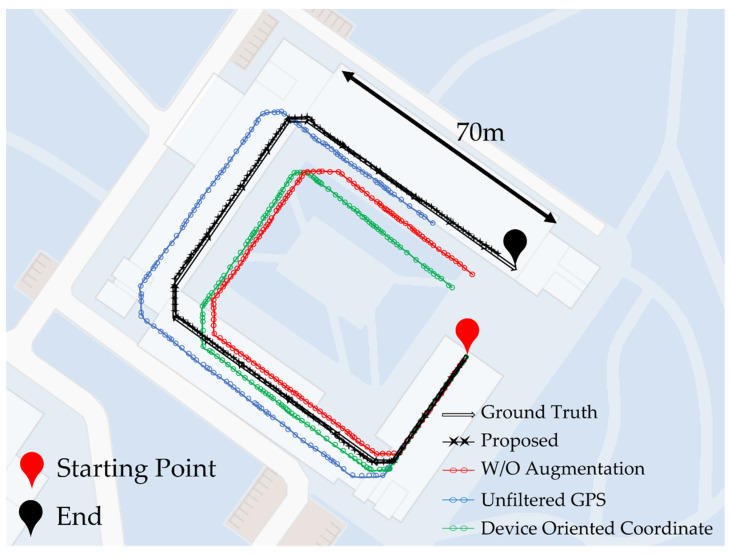
Indoor localization result.

Figure 13 shows the results of the path estimation of the test dataset collected inside a horseshoe-shaped building. The black double line path represents the ground truth information, and the black path with crosses (proposed) is the result of the path estimation of the indoor localization system proposed in this paper. We collected the data, while walking through the black double line, as shown in Figure 13, and the author’s smartphone, the Samsung Galaxy s10, was used as the data collection device. In the process of collecting this data, we collected the data, while walking along a path of a total length of 240 m and conducted a total of five-time experiments. In the process of collecting this data, we collected the data, while walking along a path of a total length of 240 m and conducted a total of five time experiments. First, the estimation results of the proposed indoor location recognition system showed a positioning error rate of approximately 3 to 5 m compared to the ground truth. This is a very low error rate compared to other comparators. The blue path (unfiltered GPS) is the result of estimating the path by learning the supervised model with the data collected, regardless of the number of GPS satellites. Regardless of the number of GPS satellites, it is difficult to achieve consistency and high reliability for the collected GPS coordinates. The GPS coordinates collected even when walking at the same speed and path depend on the surrounding environment and the number of satellite signals currently available to the smartphone. The blue path in Figure 13 shows that the errors resulting from the ground truth information significantly influence the overall performance of the system. The red path (without augmentation) is the result of estimating the path by learning 40 h of data without the use of time warping into a supervised learning model. Assuming that the experiment site was a straight path, the distance between the start and end points would not be significantly different from the ground truth. However, after the first right rotation, the red route shows a very large error compared with the ground truth. This is because the deep learning model, which learned training datasets without data augmentation, did not achieve sufficient moving-speed estimation accuracy for various moving-speed labels. Thus, the red route showed unexpected results from accumulated moving speed estimation errors. The green path (device-oriented coordinates) is the result of estimating the path by learning a training dataset that has not converted the device-oriented coordinates into earth-oriented coordinates using a supervised learning model. The data collected with device-oriented coordinates had different characteristics depending on the placement of the smartphone during data collection. As mentioned in Section 2, data that have not been generalized are difficult to use as training datasets for supervised learning models because the 3-axis of the accelerometer varies depending on the tilt or placement of the smartphone. Table 5 represents the positioning error with meters for each supervised learning model that was trained by four different training datasets from a total of five time experiments.

### 4.5. Scenario

The smartphone-based indoor localization system using deep learning proposed in this study aims for more practical use by pedestrians by providing on-demand services. Pedestrians can construct a personal indoor localization system exclusively for themselves simply by walking outdoors, while using a smartphone. The virtual scenarios for constructing pedestrian-individual systems are as follows.

Accelerometers, gyroscopes, and GPS data were collected because GPS signals can be received when pedestrians go outdoors. Afterward, when pedestrians enter the indoor environment from the outside and cannot receive GPS signals, data collection is temporarily stopped, but the data collected till then are stored in their smartphones. At this time, we assume that the collected data are valid only when there are nine or more satellite signals that can be received by smartphones, and we exclude the data when there are fewer than nine. Then, the accelerometer and gyroscope data are input into the t-SNE unsupervised learning model to classify the collected data into four cases. The size of the classified data is measured to determine whether the data size reached 3 h for each case. When training data of 3 h or a total of 12 h are accumulated for each case, smartphones stop collecting data outdoors permanently, reducing excessive battery consumption. After the training dataset of 12 h is augmented to a total size of 96 h through time warping, this training dataset is used to learn the LSTM-C model that estimates the pedestrian moving speed. When the learning is completed, pedestrians can check their path in real time by estimating the moving speed and heading direction using only accelerometer data and indoor game rotation vector sensors.

## 5. Conclusions

In this study, we propose a new smartphone-based indoor localization system using deep learning. The system used the following ideas to increase the practicality for pedestrians: 1. The movement speed calculated using outdoor GPS coordinates was set as the label of the training dataset. 2. A consistent training dataset regardless of the smartphone placement was constructed by converting accelerometer data to earth-oriented coordinates the 3-axis is fixed. 3. An unsupervised learning model was implemented to identify the minimum size of the training dataset in real time to minimize the battery consumption. In addition, the following concepts were proposed to increase the accuracy of estimating the moving speed: 1. Noise was removed to increase the accuracy of GPS and accelerometer data. 2. Data augmentation methods were applied to obtain a uniform distribution of moving speeds of pedestrians. 3. By implementing seven different deep learning models, the optimal deep learning model with the best moving-speed estimation performance was selected. After learning the parameters for the indoor localization system with the data collected outdoors, the proposed system showed a localization estimation error of approximately 3 to 5 m as a result of direct experimentation inside a horseshoe-shaped building compared to the test data of approximately 240 m in length. Compared to existing indoor location recognition studies, the proposed system shows a high location estimation accuracy, and the practicality for pedestrians is also very high. In the future, we plan to develop an in-building floor-recognition technology to provide 3D location information for indoor pedestrians. The three-dimensional location information also allows pedestrians to know which floor of the building they are on, so that they can receive much more diverse location-based services and identify safer evacuation routes in the event of a disaster.

## Figures and Tables

**Table 1:** Deep learning model hyperparameters.

Parameter	CNN	GRU	LSTM	C-GRU	C-LSTM	GRU-C	LSTM-C
Batch Size	128	128	128	128	128	128	128
Activision	ReLu	ReLu	ReLu	ReLu	ReLu	ReLu	ReLu
Optimizer	Adam	Adam	Adam	Adam	Adam	Adam	Adam
Learning Tate	0.001	0.001	0.001	0.001	0.001	0.001	0.001
Epochs	50	50	50	50	50	50	50
Loss Function	Categorical Cross entropy	Categorical Cross entropy	Categorical Cross entropy	Categorical Cross entropy	Categorical Cross entropy	Categorical Cross entropy	Categorical Cross entropy

**Table 2:** Accuracy of deep learning model for data of 12 h.

Models	Evaluation Parameters				
	Distance Error	Accuracy	Precision	Recall	F-1 Score
CNN	11.87	0.908	0.895	0.886	0.890
GRU	9.39	0.919	0.915	0.907	0.911
LSTM	9.39	0.920	0.916	0.909	0.912
C-GRU	9.12	0.931	0.926	0.918	0.922
C-LSTM	8.99	0.935	0.924	0.913	0.918
CRU-C	5.56	0.940	0.932	0.928	0.930
LSTM-C	7.39	0.956	0.950	0.944	0.947

**Table 3:** Moving speed estimation accuracy of LSTM-C model according to data size.

TW Class	Data Size (Hours)				
	4	8	12	16	20
1	0.125	0.289	0.597	0.692	0.862
3	0.439	0.804	0.889	0.944	0.951
5	0.669	0.923	0.950	0.953	0.957
7	0.832	0.938	0.953	0.954	0.957
9	0.931	0.945	0.956	0.956	0.958
11	0.931	0.949	0.956	0.963	0.969
13	0.935	0.952	0.961	0.968	0.971

**Table 4:** Parameters for each training dataset.

Parameter	Proposed	Without Augmentation	Unfiltered GPS	Device Oriented Coordinate
Filtered GPS	O	O	X	O
With Augmentation	O	X	O	O
Earth Oriented Coordinate	O	O	O	X

**Table 5:** Positioning error for each method at every epoch.

Training Dataset	Epoch (Number of Experiments)				
	1	2	3	4	5
Proposed	3.45 m	3.39 m	3.25 m	3.42 m	4.95 m
Without Augmentation	8.72 m	8.12 m	10.05 m	9.42 m	10.15 m
Unfiltered GPS	13.55 m	15.89 m	12.42 m	14.23 m	15.21 m
Device Oriented Coordinate	9.55 m	8.91 m	8.67 m	10.23 m	10.82 m

## Data Availability

Not applicable.
